# Abstract of the Proceedings of the Buffalo Medical Association

**Published:** 1870-06

**Authors:** 


BUFFALO

### Medical and Surgical Journal.

VOL. IX. JUNE, 1870. No. 11.

### Original Communications.

ART. I.—*Abstract of the Proceedings of the Buffalo Medical Association.*

TUESDAY EVENING, *June* 7, 1870.

The meeting was called to order by the President.

Members present—Drs. White, Rochester, Samo, Wetmore, Gould, Diehl, Potter, Daggett, Burke, Phelps.

Dr. Diehl, treasurer, presented the janitor's bill for care of rooms, and recommended payment of the bill, which was adopted.

At the previous meeting of the association Dr. Wetmore, on assuming the duties of President for the ensuing year, remarked that

He felt it incumbent upon him to impress upon the minds of the members of the association, that if we wished to be successful in any undertaking *we must be diligent.*

It was a trite though true saying, that anything that was worth doing at all, was worth *well* doing.

That we met here monthly for the purpose of an interchange of ideas and opinions for the purpose of discussing subjects which would the better enable us to combat the maladies with which we have to contend, for the purpose of obtaining and disseminating knowledge, and reaping from the field of science that which had been cultivated by our predecessors. That a goodly number of us were young men, and ought to be energetic, diligent, and eager to prehend the products of experienced minds. That a tendency to delinquency had always more or less existed, and wished that we might resolve to be more punctual, and make our Association a society of culture, pleasure, and discipline, each and every one striving to do something that would add to the granary of medical literature. That pathalogically our interviews might be made more interesting and instructive. That we all have opportunities more or less to present specimens of morbid anatomy, diseased structures, and anomalous growths. And if a little thought be given these subjects, and our ideas advanced for discussion, that we would soon become interested as a "Pathological Society," and be adding to the storehouse of knowledge, and learn to think that our consultations were *luxurious necessities.*

He wished that every member might be awakened to his duty. That the medical world might know, through our *valuable Journal*, that the Buffalo Medical Association was not in the arms of morpheus.

Dr. Rochester reported Hooping Cough and Scarlatina as prevailing. The Hooping Cough is malignant, and very severe and attended with the characteristics of blood poisoning, the offensive smell from the body of the patient being very marked, together with quick pulse, dry tongue, and other indications of great prostration. These symptoms indicate blood poisoning rather than disease of the nervous centers or pulmonary tract. This view is supported by West and Aitkin in their treatise.

In the treatment of this form of Hooping Cough, nutriments and stimlants should be earlier employed than is usually the practice. Bromide of potassium is a valuable remedy, and anodyne, are necessary. Dr. R. also remarked, that, being a blood poison, it was frequently communicated; and, in answer to a question by Dr. Samo, said that it was sometimes repeated in the same person.

Dr. White remarked that he was treating a case of Hooping Cough similar to the above, and had seen a case of it in a person twenty years of age; and also, but rarely, in those of advanced years.

Dr. Samo mentioned the case of a boy twelve years of age, who was suffering from a second attack.

Dr. White reported the following:

Remarkable case of Hydatid Cysts of the Liver: Mrs. T., of Lockport, N. Y.; a very large and fleshy woman, was the mother of three children, and had suffered two miscarriages. In 1858, twelve years ago, and following her last confinement, a tumor was discovered in the upper part of the right lumbar region. In 1863, after an abortion, her abdomen became greatly distended with fluid, and in May, 1863, she was tapped,—sixty-five pounds of a serous straw-colored fluid being drawn off. After the evacution of this fluid the old tumor, in the right side, could be felt. The medical gentleman who tapped her thought that the fluid was contained in an ovarian cyst; others, however, thought that the tumor was hepatic in origin, and that the cysts, if there were any, were likewise connected with the liver, but that the fluid was ascitic. After this operation the patient remained comfortable for several months. In May, 1864, her abdomen again became enlarged. She was again tapped, and thirty-two pounds of a darker colored fluid drawn off. This was followed by another tapping in September, 1864. She then became very comfortable, performed several journeys, attended to her household duties, and herself forgot the tumor in her side. Her health was good from September, 1864, to March, 1869. Her attending physician, Dr. Leonard, however, thinks that the tumor could at any time during this long interval of health, be clearly defined. He had had two or more occasions between September, 1864, to March, 1869, for examining the abdomen, and could define the tumor, although its existence gave rise to no inconvenience on the part of the patient. In March, 1869, her health became again impaired, and her abdomen again became distended. She was, therefore, again tapped, removing thirty-three pounds of fluid. At this tapping the fluid was not uniform either in consistence or color; some of it was transparent, some of it coffee-colored, some of it serous, and some of it much thicker than serum.

In September, 1869, she was again tapped, and from that time to May, 1870, the operation was repeated, I believe, four times,—little fluid escaping either time. The abdomen had now become greatly enlarged, and I saw her May 9, 1870, in consultation with Dr. Leonard. Her abdomen was terribly distended, above the umbilicus, with a hard unyielding mass; but below the umbilicus, fluctuation was distinct. The gelatinous quality of the fluid drawn off by an operation performed a few days previous to my visit, its small amount, only six pounds escaping; the fact that an elastic catheter had been passed in its whole length after removal of the trochar and, withal, the great duration of the disease, led me to hope that the fluid in the lower portion of the abdomen was contained in ovarian cysts, and that although there might be hepatic disease present, there probably was also multilocular ovarian disease. The symptoms were urgent, the discomfort insupportable, and as a last resort I advised to cut down upon the cysts, and if, upon exploration, it was deemed practicable, to remove them. Upon reflection the patient and her friends concluded to take the risk, and adopt the course I had recommended. Accordingly on the seventeenth day of May, in presence of Drs. Leonard, McCollum, Gould, Palmer, Foote, Kittinger, and others of Lockport, and assisted by Dr. Potter, of Buffalo, all concurring with me in the expediency of the course proposed, I cut down through the parietes of the abdomen. Upon opening the abdominal cavity there was found, opposite and above the umbilicus, a large irregular morbid fleshy growth extending across the abdominal cavity. Below this, and a little to the right of the median line of the body, there was a large cyst filled with fluid. The incision through the abdominal parietes was then enlarged in a downward direction, the cyst tapped, and as nearly as could be estimated forty pounds of offensive fluid, looking very much like gruel, drawn off. The sac, containing this fluid, was found to have its pedicle above and attached to the lower margin of the enlarged right lobe of the liver throughout its whole extent. A portion of the interior of this cyst looked like the walls of an abscess, showing that either this limited portion of the cyst had become a pus secreting surface, or that decomposition had already begun to take place in the walls of the sac, and likewise, accounting for the offensive odor of the fluid contained in it. After removing everything which could be safely taken away, the wound was closed in the usual manner for ovariotomy. The patient had been kept under the influence of anæsthetics for about thirty minutes. She was now given stimulants and an anodyne, and carried to her bed. She did not, however, rally, and although she became so intelligent as to converse with her friends, she sank and died six hours after the conclusion of the operation.

*Post mortem*, two hours later. Abdominal cavity containing no blood. Liver immensely enlarged, and filled with cysts containing fluid of different colors and degrees of consistence. The whole organ, with the exception of a small portion of the left lobe, had no appearance of liver, being rather an agglutination of degenerate growths, in which were interspersed cysts of various sizes. The stomach, duodenum and jejunum, were inclosed in the mass, so that it was necessary to cut the intestine in order to remove them. The weight of this liver, with the remaining smaller cysts, was twenty-three and one-fourth ($23\frac{1}{4}$) pounds. The weight of the fluid removed by the operation just described, was forty pounds. Now, allowing that four-fifths ($\frac{4}{5}$) only of this fluid came from the larger cyst,—in other words, that one-fifth of it was blood and ascitic fluid, we would then have as a low estimate for the weight of this liver with its cysts, fifty-five and one-quarter ($55\frac{1}{4}$) pounds, about fourteen times its normal weight. The uterus and ovaries were normal in size, position, and appearance.

Diagnosis: Hydatid cysts of the liver: echinococci of Frerichs.

I have taken the liberty to report this case, gentlemen, because hydatid cysts of the liver are very infrequently met with by American practitioners, and because I can find no record anywhere of a liver acquiring anything like the dimensions here described. It is, I think, unparalleled in size.

At the conclusion of this report Dr. White gave a brief resume of the nature, clinical history, pathological history, symptoms, and treatment, both medical and surgical, of hydatid cysts of the liver.

Dr. Diehl was requested to prepare his paper on post mortem appearances for the August meeting of the association.

Dr. Samo stated that his paper would be ready for the next meeting in July.

The President, Dr. Wetmore, having called Dr. Rochester to the chair, remarked:

That the regular essayist not having had time to favor us, that he would, by his request and the permission of the Association, read a poem entitled "*Medical Ethics.*" Although it was an anomalous way of expressing one's ideas in a medical society, it would nevertheless be a change. Variety, however, is not always expected to be spicy. But inasmuch as it was not designed for publication, nor to establish a novelty in expression, he hoped they would bear with his eccentricity.

### Medical Ethics.

No captious thoughts, or aim, or disposition,
Nor wish, nor jealous feeling, would
In this hasty, hurried manuscript
Bear intolerance, by he who writes suggestions
Only, on such a theme as etiquette.

That medical ethics
Needs reform, does not admit of cavil, nor of doubt,
And needs that culture long ignored
By some whose lives of earnest real endeavors
Of firm, unwavering tread, of good and noble purpose
Have made them true philanthropists.

Though philanthropic in their aim,
Like nature everywhere steals in those selfish
Hopes, and fears, and makes them jealous creatures:
Could they forbear incentives and keep aloof
The master passion, *loss and gain*, 't would be
The *golden rule*, instead its quite too often *rule or ruin.*

Gold and fame o'erpowers the amateur,
But rides a king triumphant through the world,
Makes joy and sunshine everywhere, fills hearts
With glee, with hope, and cheer, with mirth
And pride, and "life that's real,"
But not beyond the grave.

The body politic, all professions in life,
Commerce and trade, the same pseudo ethics
Portray. Being governed by habit: custom
And fashion. More potent than law;
Of human duty at least. Thus
The ruling power of wealth and fame;
Becomes established, and keeps at bay,
The young of our profession.

Full well do I remember
While in my youthful days, was on a time
Selecting works of art and science, books
Histories, lives of men like Scott and Moore,
And glancing o'er a preface page, I read

"Like ivy 'round the oak the *poor*
Young man must cling around some wealthy autocrat,
Some power above his humble parentage,
Some one whose fame o'erspreads the vast
Domain of rich and poor, of high and low;
Of pomp and pride, or else his progress
Would be slow in gaining reputation.
And even then his efforts might be
Thwarted."

Incensed, indignant as I was
Threw down the book; and left the huckster
Just as poor as when I entered
Little thinking that his loss, was also mine;
That on those pensive pages, reasons why
Were penned.

Since then I've pondered o'er
And o'er a thousand times or more its meaning
And all at once 'twas rendered.

I sought the book,
And found my musings verified.
Perverted science was the theme,
And that of human duty.
In other words 'twas ethics that demurred
And stayed, and kept in check
The would be energetic.

No little truth was there evinced,
Which custom makes a law:
Ignoring that which nature gave,
For that which wealth would draw.
Opprobrious custom in extreme
Secured—perhaps by letters patent—
A thousand years or more ago
'Twere better were it latent.

Another reason has its sway, aside from power
In wealth, and fame, and reputation,
Is recognized in young and old, in high and low
All o'er the world, in every station.

'Tis that of jealousy.
Of fear, which fostering fancy feels,
And fills the soul, the mind, the will,
With false impressions.

Unpensive selfish
Creatures, unsocial in their manners,
Uncordial in their greeting, morose and peevish
Keep aloof, that which should make them happy.

Methinks I've met with just such men
In our profession. And with their jealous notions
Had great opinions of themselves. Could ne'er

Be taught or cultured. Ignored the young
And those of less experience. And here
I'll detail one such case.

Once on a time
Was summoned by a friend to see another's
Patient. Responded no! Unless with the attendant.
"But you are wanted," he replied, "to take the case
Entire." Can't help it sir. There's Doctor A ——,
Much older in the profession, who cares for him
I cannot doubt, much better than could I.

But all my reasonings futile proved.
At last I did agree to meet the doctor the
Following day for consultation,
The hour came, and I was there;
Another passed, and I in waiting still:
Began to think of leaving. But in my exit
Met the sage, with a good morning doctor,
Shaking hands. A fine nice morning this?
"*Yes*," was his reply. "*Have you seen the patient, sir?*"

Seen the patient? No!
Do you suppose
That I, ignoring ethics would comply
With friends, and treat you so unkindly?

He turning to the patient's friend, remarked
"*If you'r dissatisfied with me, let him see the patient.*
*Go in and see him, sir; I'll ne'er prescribe*
*For him again.*"

I'll see him with you
Was my reply. But not to take the patient.
I came for consultation.

"*Come in, come in,*
*Here's the patient, who's been sick three*
*Weeks or more, with hepatitis.*"
Detailing here prescriptions, then remarked
"*He's doing well, and I'll continue just the same.*"
Not asking me to approach the couch
To even see the patient, much less to make
Suggestions. But I saw the sick man
And remarked: Be patient, sir, and you'll
Get well. The doctor's doing all that can be done
By any one. Keep quiet as you can,

The doctor gruffly muttered out
" *Well, that will help him some, perhaps,*
*That must be consolation.*"

I took my hat and walked away
Dismayed, disgusted, and disposed to think
'Twas *modern consultation.*

The patient was an honest man,
And did not like such play;
He summoned me just once again,
No ethics in the way.

The true gentleman has true etiquette
Is true to himself, his neighbor and his profession,
Is noble, just and kind, honorable in his dealing,
Conscientious in his aim, social in his nature,
Cordial in his greeting, obliging to his friends,
Is genial and refined; and honor, and distinction,
Will crown his efforts everywhere.

Sir John Herschell says,
" If a man has desire, and disposition
To become a scholar, and a gentleman of culture,
Give him discipline, books, and opportunity,
And success will crown his efforts,
Unless, perchance, his ethics become perverted."

'Twould seem that Herschell had in view
The power behind the throne, that always has,
And always will predominate. As long as custom
Makes a law, and envy strife, and jealousy,
Is not in abeyance held.

'Twas then, as now, the o'erpowering
Conquering ruling force. The stay of opportunity,
The one great secret of *unsuccess.*

Of unpropitious futile efforts.
Of blasted hopes and energies.

Give the young an opportunity, and good discipline;
Support their earnest, real endeavors. Buoy up their
Anxious, pensive, fettered powers. And put the shoulder
To the wheel when flagging 'tis observed.
And the primeval base of the *golden rule*

Will have been established. Thus,
The flowers and prints of literature,
The confiding, buoyant, fraternal power,
And the guiding stars of etiquette,
Will be thickly strewn along the path of life
To cheer the pilgrim on, as he journeys
O'er the hills, and through the vales
Of time.

The first lessons in life
Should be truthfulness to ourselves. Conscientiousness
Should guide our footsteps; should be the landmark,
The beacon light, as well as the incentive
Of all our future actions. Then wisdom
Truthfulness, prosperity, and goodness,
Will predominate.

Goodness,
Like all virtuous blessings, necessarily involves
A purity of purpose; a principle and desire
To avoid error, and all flagitious incentives.
It teaches us the "*Will do right*," motto, without which
We are constantly subjecting ourselves to the
Incredulity of our fellow men, and associates.

The confidence of our friends, our patrons,
And the world, is unestablished,
Upon which we build
A reputation, and distinction; the utility of which
Is indispensable to become great in our profession,
And honorable servants to the people.

If we are true to ourselves
We will conscientiously be true to others, and thus
True ethics will be established. Guiding
Our frail rudder by the old motto,
"*We will do right*," we will be enabled in drifting
Down the tide of life, to evade the billows of despair,
The crags of wantonness and disappointment.

Our wayside thoughts
May err. Our transient and erratic minds
May linger o'er the driftwood, scum, and dross,
Of a wayward world. And at times
'Twill seem the o'erpowering surf
In its retreat, will take us with it.
Saddened and disappointed in business

And affairs of life, we know not
What to do, where to go, or where to wander,
To drive dull care away.
And thus, we are apt to yield
To the allurements, vices, and errors
Of perverted, depraved, and enfeebled minds.

But like the retreating forces
Of a despondent army, by good generalship
We are sure to rally and call to aid
The *old motto*. And all is well,
The victory is won, the battle o'er the enemy ours.

Without these qualifications and endowments
Acquired by our own will, desire, effort and persistence,
We are but little better than the uncultivated native,
Devoid of self respect, principle, honor, or distinction;
And subject to disappointment in confidence,
In life, in business, in friends, and in the future.

Keeping in mind then,
That conscientiousness is the goal of goodness,
And goodness and purity the principle of the *golden rule*,
And the motto of *right* our beacon light,
We will be enabled to "paddle our fragile bark"
Through the billows of adversity, despondency, and despair,
And gain the shore of distinction and honor;
True to ourselves, our neighbor, and our God.

Adjourned.

